# Effect of Ag on Properties, Microstructure, and Thermostability of Cu–Cr Alloy

**DOI:** 10.3390/ma13235386

**Published:** 2020-11-27

**Authors:** Yuqing Sun, Gaolei Xu, Xue Feng, Lijun Peng, Guojie Huang, Haofeng Xie, Xujun Mi, Xinhua Liu

**Affiliations:** 1 State Key Laboratory of Nonferrous Metals and Processes, GRIMAT Group Co., Ltd., Beijing 100088, China; 13521565625@163.com (Y.S.); xie.haofeng@126.com (H.X.); sklcopper1967@163.com (X.M.); 2 Key Laboratory for Advanced Materials Processing (MOE), University of Science and Technology Beijing, Beijing 100083, China; liuxinhua18@163.com; 3 GRIMAT Engineering Institute Co., Ltd., Beijing 101407, China; 4 General Research Institute for Nonferrous Metals, Beijing 100088, China; xugaolei@126.com; 5 Zhejiang Libo Industrial Co., Ltd., Shaoxing 312050, China; 6 Beijing Laboratory of Metallic Materials and Processing for Modern Transportation, Beijing 100083, China; 7 Beijing Advanced Innovation Center for Materials Genome Engineering, University of Science and Technology Beijing, Beijing 100083, China

**Keywords:** Cu–Cr system alloy, aging process, microstructure, thermal stability, physical properties

## Abstract

Cu–Cr-based alloys exhibit excellent electrical conductivity and strength, but their poor thermal stability limits their application in industry. In this paper, Cu–0.2Cr (at. %) and Cu–0.2Cr–0.12Ag (at. %) alloys were prepared to study the effect of Ag on the properties, microstructure, and thermal stability of the Cu–Cr alloy. Microstructure and precipitation were observed by an optical microscope (OM) and a transmission–electron microscope (TEM). After cold-drawing by 99.9% and aging at 450 °C for 2 h, the peak hardness and electric conductivity of the Cu–Cr alloy were 120.3 HV and 99.5% IACS, respectively, and those of the Cu–Cr–Ag alloy were 135.8 HV and 98.3% IACS, respectively. The softening temperature of the Cu–Cr alloy was 500~525 °C, and that of the Cu–Cr–Ag alloy was about 550 °C. The creep strains of the Cu–Cr and Cu–Cr–Ag alloys at 40 MPa and 400 ℃ for 50 h were 0.18% and 0.05%, respectively. Ag elements improved the thermal stability of the Cu–Cr alloy. Recovery and recrystallization occurred before the coarsening of precipitates during the softening process. Ag atoms mainly improved the softening resistance of the alloy by delaying recrystallization, and mainly increased creep resistance by preventing the increase in mobile-dislocation density.

## 1. Introduction

Cu–Cr-based alloys are typical precipitation-strengthening alloys with good electrical conductivity and mechanical properties, and they are used in integrated–circuit lead frames, connectors, and electrical equipment [1,2,3,4,5]. These alloys include Cu–Cr–Zr [6], Cu–Cr–Zr–Mg [7], Cu–Cr–Ag [8], and Cu–Cr–Mg [9]. The electronic and crystal structures of Ag atoms are like those of Cu; thus, the conductivity of the alloy decreases only slightly. In addition, a small number of Ag elements are readily soluble in the Cu matrix under atmospheric smelting [10], and lead to obvious solid-solution strengthening [11] and precipitation strengthening [12], so the Cu–Cr–Ag alloy interests many researchers.

Liu et al. [8] reported that the strength and conductivity of the Cu–0.13Cr–0.074Ag alloy were 473 MPa and 94.5% IACS, respectively, after solution-treating at 870 °C for 1 h, cold rolling (CR) by 60%, and aging at 480 °C for 2 h. The Ag element was uniformly distributed in the Cu matrix. The structure of Cr phases in the alloy was transformed from face-centered cubic (fcc) to body-centered cubic (bcc), and the orientation relationship between Cr precipitates and matrix was transformed from cube-on-cube to N–W. Xu et al. [11] determined that the optimal content of Ag in the Cu–0.91Cr alloy was 0.11 wt % according to the properties of the alloy and the price of silver. Insoluble Cr phases during casting were decreased and spheroidized with increasing Ag content, leading to better alloy properties. Islamgaliev et al. [12] improved the tensile strength of the Cu–0.5Cr–0.12Ag–0.06Fe–0.06P–0.05Si alloy to 845 MPa through severe plastic deformation, and electrical conductivity remained at 81% IACS. Yuan et al. [13] confirmed that dynamic recrystallization [14,15] occurred in the Cu–Cr–Ag alloy during updrawing continuous casting, and average grain size decreased, thus enhancing the mechanical properties of the alloy. Watanabe et al. [16] confirmed that 0.1 wt % Ag reduced the interprecipitate spacing of the Cr phase in the Cu–0.5Cr alloy and inhibited recovery during aging, thus improving strength. To summarize, studies on the Cu–Cr–Ag alloy mainly focused on the preparation process, mechanical properties, and microstructure, but less on thermal stability. Mahmadi et al. [17] studied the creep properties of the Cu–0.3Cr–0.1Ag alloy in the aging and deformation states, and reported that the aging alloy showed better creep resistance due to more Cr precipitates, but the creep process of the Cu–Cr alloy affected by the Ag element was not explored. Yuan et al. [18] determined that the softening temperature of the Cu–0.19Cr–0.08Ag alloy was 550~600 ℃, which was ascribed to the Cr precipitates impeding the movement of dislocation. However, the softening mechanism of the alloy and how Ag affected the softening process of the Cu–Cr alloy were not clear. Therefore, it was necessary to explore the effect of Ag on the microstructure, mechanical properties, and thermal stability of the Cu–Cr alloy.

In this paper, the mechanical properties, softening properties, and creep properties of Cu–0.2Cr (at. %) and Cu–0.2Cr–0.12Ag (at. %) were tested. Microstructure evolution during the softening and creep process was analyzed, and the mechanism of Ag on the mechanical properties and thermal stability of the Cu–Cr alloy was explored.

## 2. Materials and Methods 

Table 1 lists the chemical compositions of the alloys in this study. Raw materials were composed of a 99.99% high-purity cathode copper Cu–5%Cr (wt %) master alloy and pure silver. Materials were melted at 1200 °C in an argon atmosphere by a ZG0025 vacuum medium-frequency induction furnace (Shanghai Chenhua Technology Co., Ltd., Shanghai, China) and homogenized at 900 °C for 12 h followed by water quenching. The homogenized samples were cold-drawn by 99.9% deformation and aged at 450 °C for different times to determine the best comprehensive properties. After peak aging treatment, the samples were annealed at 300~700 ℃ for 1 h to measure the softening temperature of the Cu–Cr and Cu–Cr–Ag alloys. The creep properties of the alloys were tested at 400 and 500 °C for 50 h with a load of 40 MPa.

Hardness was measured by a 430 SVD-type Vickers hardness tester (Beijing Huahai Henghui Technology Co., Ltd., Beijing, China) with 5 kg loading and a holding time of 15 s. Electrical conductivity was tested by a Sigma 2008 digital eddy current conductivity meter with 500 KHz. Each value was measured 5 times, and the average value was taken. Creep properties were measured by an RD-50 high-temperature creep-testing machine. The microstructure and precipitates of the alloys were observed by an Axiovert 200MAT (Guangdong Jingpu Industrial Co., Ltd., Guangdong, China) optical microscope (OM) and FEI Tecnai G^2^ F20 (FEI, Hillsboro, OR, USA) electronic microscope. The samples for transmission-electron-microscope (TEM) observation were prepared by electrolytic polishing; the ratio of nitric acid to methanol was 1:4.

## 3. Results

### 3.1. Properties

#### 3.1.1. Mechanical Properties

**Figure 1:**
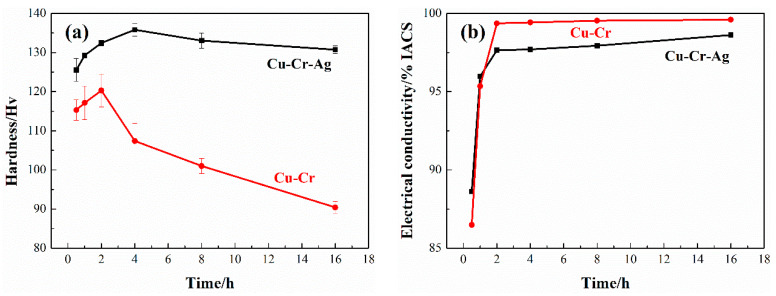
(**a**) Hardness and (**b**) electrical conductivity of alloys at 450 °C.

Figure 1 shows the hardness and conductivity of the Cu–Cr and Cu–Cr–Ag alloys at 450 °C. As shown in Figure 1a, compared with Cu–Cr, the Cu–Cr–Ag alloy showed higher hardness at the same aging time. The hardness of the two alloys increased rapidly and then decreased after reaching the peak value. The peak hardness of Cu–Cr reached 120.3 Hv after aging for 2 h, while that of the Cu–Cr–Ag alloy reached 135.8 Hv after aging for 4 h, and the decreasing trend was slower. When aging time was 16 h, the hardness of the Cu–Cr alloy was only 90 Hv, which was 30 Hv less than that at the peak aging state, and the hardness of the Cu–Cr–Ag alloy was 130 Hv, which was only 5 Hv less than that of the peak aging condition. The result illustrated that the hardness and stability of the mechanical properties of the Cu–Cr alloy improved with Ag elements. The conductivity of both alloys rapidly increased and gradually stabilized, as shown in Figure 1b. Compared with cold-rolled alloys, the conductivity of the Cu–Cr and Cu–Cr–Ag alloys aged for 2 h increased by 13.1% and 9.8% IACS, respectively. Electrical conductivity corresponding to the peak hardness of the Cu–Cr and Cu–Cr–Ag alloys was 99.5% and 98.3% IACS, respectively.

#### 3.1.2. Softening Resistance

**Figure 2:**
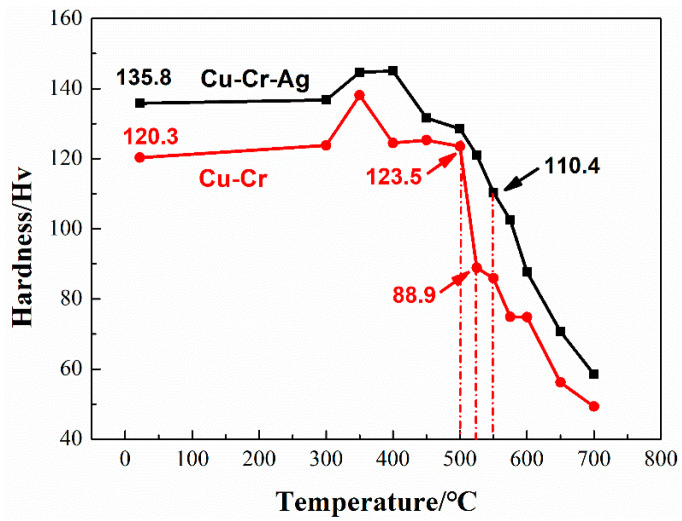
Hardness of test alloys at different annealing temperature levels for 1 h.

Figure 2 presents the change in hardness of the alloys after 1 h incubation at different annealing temperatures. The hardness of the two alloys slightly increased and then decreased with the rise of temperature. According to GB/T 33370-2016, the softening temperature of copper and copper alloys is defined as the temperature corresponding to hardness dropping to 80% of the original hardness. The hardness of the Cu–Cr binary alloy that annealed at 500 °C was 123.5 HV, which rapidly decreased to 88.9 Hv at 550 °C, namely, 74% of the peak-aging hardness. This indicated that the softening temperature of the Cu–Cr alloy was 500~525 °C. The hardness of the Cu–Cr–Ag alloy at 550 °C was 110.4 HV, which is about 80% of the peak-aging hardness. Therefore, softening temperature was about 550 °C, which was higher than that of the Cu–Cr alloy.

#### 3.1.3. Creep Resistance

**Figure 3:**
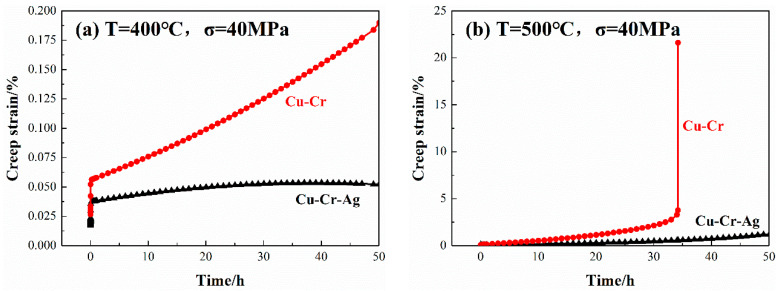
Creep curves of alloys at (**a**) 400 and (**b**) 500 °C under 40 MPa stress.

The creep curves of both alloys at 400 and 500 °C are shown in Figure 3. Under the same loading stress, the creep curves of the two alloys showed the same trend, namely, strain positively rose with loading time. Strain also rose with an increase in temperature. The creep strain of the Cu–0.2Cr alloy was 0.18% after loading at 400 °C for 50 h, and that of the Cu–0.2Cr–0.12Ag alloy was 0.05%. However, the creep strain of the Cu–Cr alloy obviously changed as temperature rose to 500 °C. When loading time was increased to 35 h, a creep fracture occurred in the Cu–Cr alloy, but the creep stress of the Cu–Cr–Ag alloy was only 1.3% after 50 h. Data illustrated that the Ag element improved the thermal stability of the Cu–0.2Cr alloy, which was consistent with the results in Figure 2.

### 3.2. Microstructure

**Figure 4:**
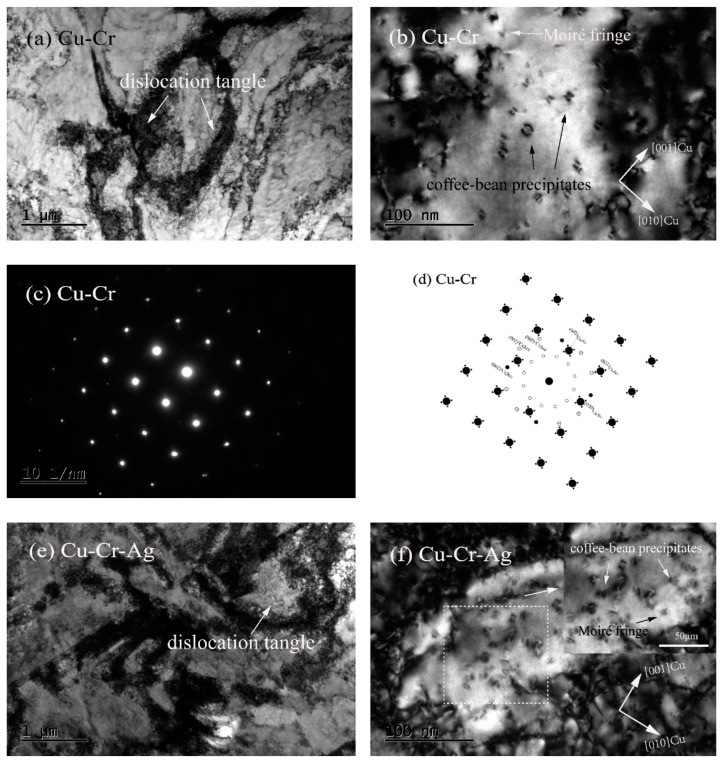
Morphologies and selected-area electron-diffraction (SAED) pattern of peak-aged alloys. (**a**,**b**) Bright-field image of Cu–Cr alloy with zone axis of (001)_Cu_; (**c**) SAED pattern of (**b**); (**d**) schematic diagram of (**c**); (**e**,**f**) bright-field image of Cu–Cr alloy with zone axis of (001)_Cu_.

Figure 4 shows the morphologies and selected-area electron-diffraction (SAED) pattern of the peak-aged alloys. Figure 4a shows that high-density dislocations formed in the Cu–Cr alloy that displayed a morphology of mutual entanglement. In addition, many dispersed nanoscale-precipitate phases existed in the Cu matrix, and their morphologies included coffee-bean contrast and Moiré fringe contrast, as presented in Figure 4b. According to previous studies, the precipitate with coffee-bean contrast was a Cr phase with fcc structure [19,20,21,22]. Figure 4c,d show the selected-area diffraction-electron and schematic diagrams of the {001}_Cu_ direction, respectively. Two sets of diffraction spots occurred near the Cu matrix spots. One set was the diffraction of 0°, 30°, and 60° to the <011>_Cu_ direction of the matrix, and the other was a circle of diffraction spots near the center. According to previous studies [23,24], diffraction spots around {022}_Cu_ in the matrix were the Cr phase with a bcc structure. Therefore, precipitates with Moiré fringe contrast were bcc Cr, according to the morphology characteristics and distribution orientation of precipitates in Figure 4b. The Moiré contrast was caused by the superposition of the precipitates and double diffraction between precipitate phase and matrix [19,25]. The orientation relationship with <001>_Cr_‖<110>_Cu_ existed between Cr phase and matrix, namely, an N–W orientation relationship. Many intertwined dislocations were found in the Cu–Cr–Ag alloy, as shown in Figure 4e, which were the same in the Cu–Cr alloy. Compared with Figure 4b,f, Ag elements did not change the microstructure of Cr precipitates, but inhibited the growth of Cr precipitates.

**Figure 5:**
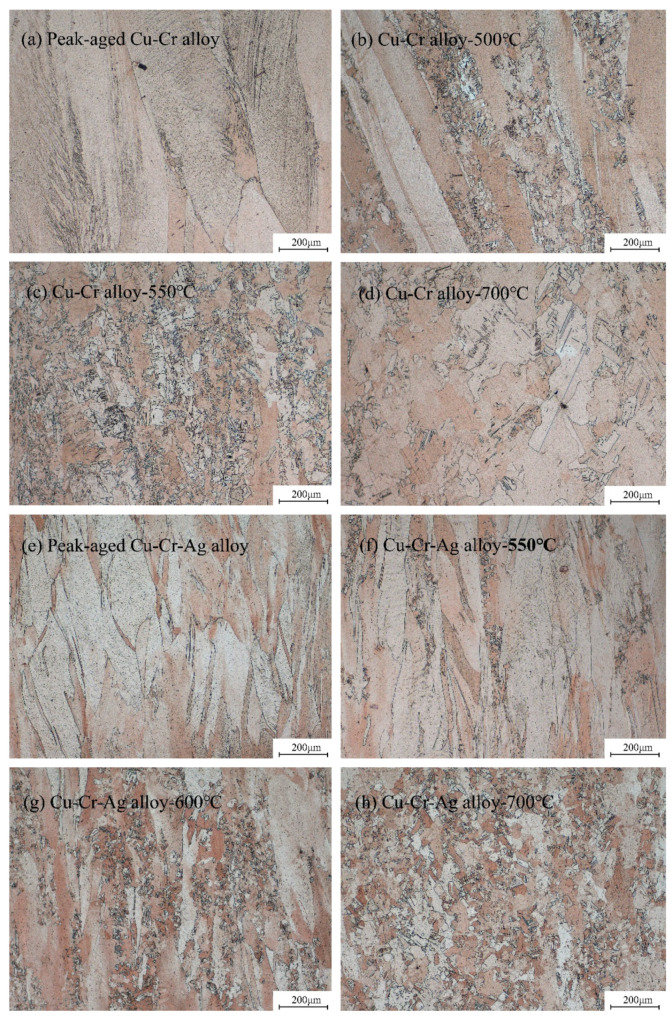
Microstructures of (**a**–**d**) Cu–Cr and (**e**–**h**) Cu–Cr–Ag alloys annealed at different temperature levels for 1 h.

Figure 5 shows the microstructure of alloys after annealing at different temperature levels for 1 h. As shown in Figure 5a,e, the fiber structures existed after large plastic drawing deformation of the alloys. Recrystallization occurred in the Cu–Cr alloy at 500 °C, as shown in Figure 5b, which was accelerated with rising annealing temperature, and the fiber structure eventually transformed into a recrystallized equiaxed structure, as shown in Figure 5d. The microstructure evolution of the Cu–Cr–Ag alloy during annealing was similar to that of the Cu–Cr alloy, but Ag elements obviously inhibited the recrystallization of the alloy, which corresponded to the results in Figure 2.

**Figure 6:**
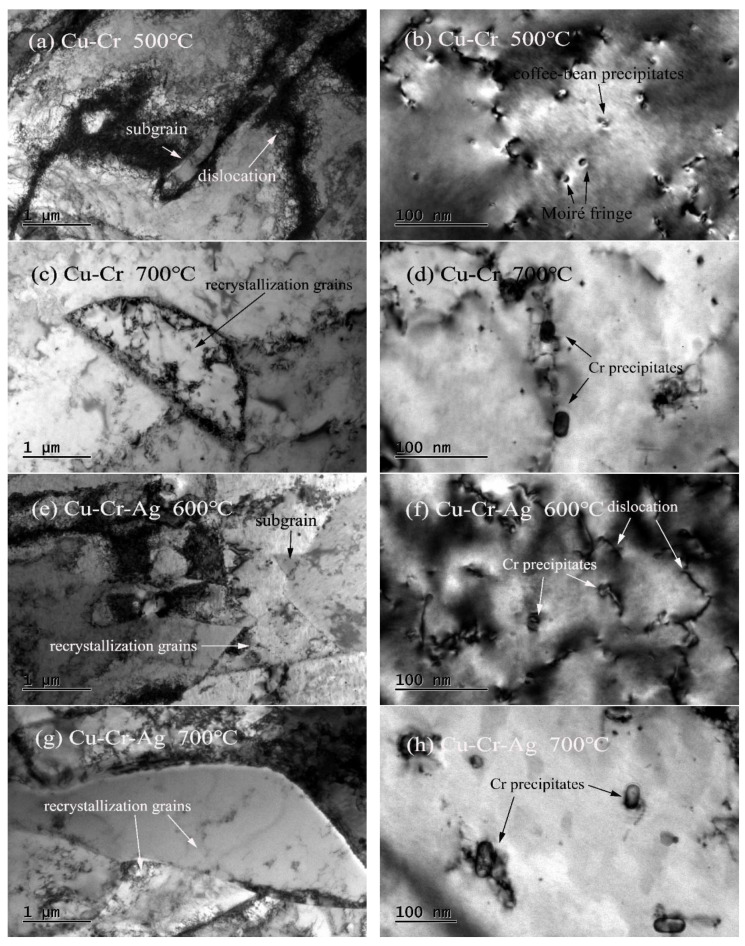
Microstructures of both alloys after holding for 1 h at different annealing temperature levels. Bright-field images of (**a**–**d**) Cu–Cr and (**e**–**h**) Cu–Cr–Ag alloys.

The TEM microstructures of both alloys at different annealing temperature levels for 1 h are shown in Figure 6. Subgrains, and fine and dispersed Cr precipitates were found in the Cu–0.2Cr alloy at 500 °C, as shown in Figure 6a,b, and the size of the precipitated phase was consistent with that in the peak aging state. As temperature rose to 700 °C, dislocation density decreased, obvious recrystallized grains existed in the Cu–Cr matrix, and precipitates coarsened, as shown in Figure 6c,d. Recrystallization of the Cu–Cr–Ag alloy occurred at 600 °C. The size of Cr precipitates did not significantly increase, but recrystallized grains grew, and precipitates obviously coarsened at 700 °C. Therefore, the softening of the Cu–Cr and Cu–Cr–Ag alloys was accompanied by the recovery, recrystallization, grain growth, and coarsening of precipitates.

**Figure 7:**
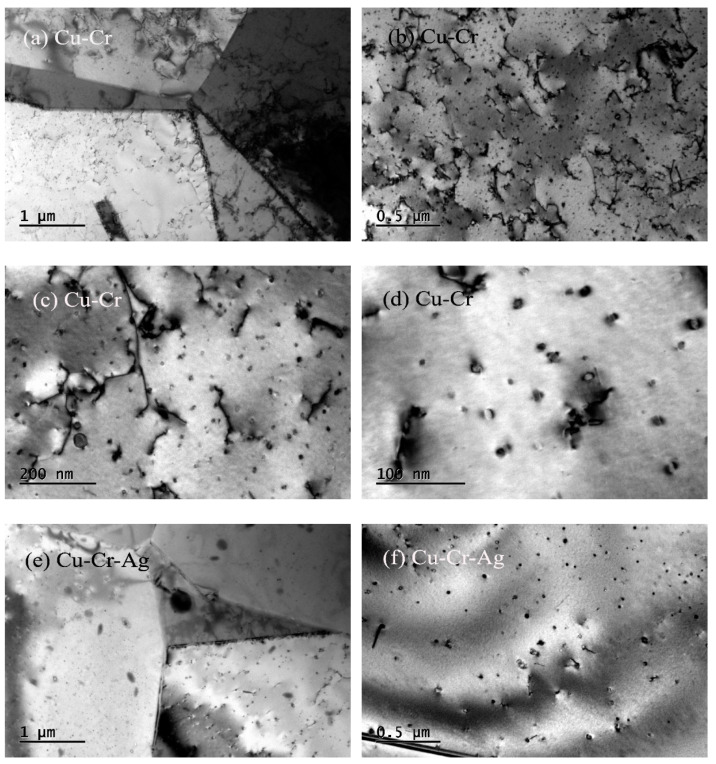

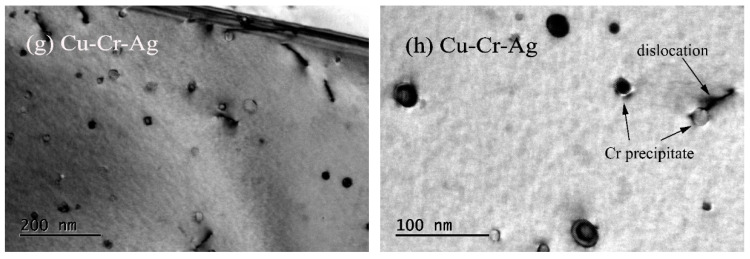
Microstructures of alloys after creep test at 500 °C. Bright-field images of (**a**–**d**) Cu–Cr and (**e**–**h**) Cu–Cr–Ag alloys.

Figure 7 shows the microstructures of both alloys after a creep test at 500 °C. Recrystallization was found in the Cu–Cr alloy in Figure 7a, indicating that it occurred during the creep at a stress of 40 MPa and temperature of 500 °C. Meanwhile, dislocations were rearranged during the creep formed subgrain boundaries and absorbed by grain boundaries, which greatly reduced dislocation density in the matrix. Many precipitates existed in the matrix, which obviously interacted with the dislocations, as shown in Figure 7b,c. Precipitates can be divided into two types: the original precipitates of 20 to 30 nm, and precipitates of 10 to 15 nm that formed during creep process. Creep strain increased with loading time, which promoted the movement of dislocations and the formation of new dislocations. Therefore, the interaction between dislocations and precipitates became more serious, resulting in stress concentration, the forming of crack sources, and the acceleration of the generation of creep cracks. These eventually led to the Cu–Cr alloy fracturing at 35 h. In addition, according to the morphology characteristics of the precipitates in Figure 7d, precipitates in the matrix were of bcc Cr phase with Moiré fringe contrast, indicating that the precipitate structure eventually changed from fcc to bcc during the creep of the Cu–Cr alloy. This was attributed to the fact that the creep is a thermal-activation process, so energy and structure develop in the stablest fashion. The mobile-dislocation density of the Cu–Cr–Ag alloy was significantly lower than that of the Cu–Cr alloy. The morphology characteristics of precipitates in the Cu–Cr–Ag alloy were similar to those of the Cu–Cr alloy, but average size was slightly larger.

## 4. Discussion

The hardness of the Cu–0.2Cr alloy was improved by 0.12 at. % Ag, shown in Figure 1. This was attributed to Ag elements in the matrix playing a role in solution strengthening, hindering the growth of precipitates (Figure 4), and reducing interprecipitate spacing [16]. In addition, Ag elements reduced the stacking-fault energy of the copper alloy [26] and increased dislocation density, thus enhancing the mechanical properties of the Cu–Cr alloy. The aging time of the Cu–Cr alloy reaching peak value was prolonged by Ag, which was ascribed to Ag inhibiting the growth of precipitates [27,28].

The recovery, recrystallization, and coarsening of precipitates occurred in the softening process of the test alloys, as shown in Figure 5 and Figure 6. When the alloys were annealed near the softening temperature, recovery and recrystallization occurred in the two alloys, but precipitates barely coarsened. As annealing temperature was increased to 700 °C, the diffusion rate of atoms rapidly increased [29], resulting in the precipitates of the two alloys obviously coarsening. Therefore, the recovery and recrystallization of the alloys occurred prior to precipitation coarsening during softening of the alloys. The softening resistance of the Cu–0.2Cr alloy improved by adding 0.1 at. % Ag (Figure 2), which was attributed to Ag atoms raising the recrystallization activation energy of the copper alloy [30] and delaying recrystallization during softening (Figure 5). Meanwhile, the Ag elements had little effect on the precipitates (Figure 6), indicating that recrystallization was the main factor that caused alloy softening.

The addition of 0.12 at. % Ag elements also improved the creep resistance of Cu–0.2Cr (Figure 3). The creep process was accompanied by changes in grains, dislocations, and precipitates. Recrystallization occurred in the Cu–Cr and Cu–Cr–Ag alloys after the creep test at 500 °C with a stress of 40 MPa, and the dislocation density of Cu–Cr was higher than that of the Cu–Cr–Ag alloy (Figure 6). This was attributed to complex dislocation changes during the creep. Plastic deformation caused an increment of dislocation density in early deformation, and the dislocations in different slip systems interacted to form dislocation entanglement and cells. The dislocation cells rearranged to form a subgrain boundary with the increase in creep strain. In addition, precipitates gradually coarsened during the creep, which reduced the pinning force of dislocation and resulted in the substructure evolving into a recrystallized structure. Compared with the Cu–Cr alloy, the size of precipitates in the Cu–Cr–Ag alloy was larger after the creep test, which may be attributed to Ag hindering the increase in dislocation density during creep and reducing the interaction between precipitates and dislocations.

## 5. Conclusions

This work studied the mechanical properties, thermal stability, and microstructure of the Cu–0.2Cr (at. %) and Cu–0.2Cr–0.12–Ag (at. %) alloys. Results are summarized as follows:

1. The addition of 0.12 at. % Ag increased the hardness of the Cu–0.2Cr alloy and slightly decreased electrical conductivity. The peak hardness of the Cu–0.2Cr alloy was 120.3 Hv after aging at 450 °C for 2 h, while that of the Cu–Cr–Ag alloy reached 135.8 Hv after aging at 450 °C for 4 h. Electrical conductivity corresponding to the peak hardness of the Cu–0.2Cr and Cu–0.2Cr–0.12Ag alloys was 99.5% and 98.3% IACS, respectively.
2. The softening resistance of the Cu–0.2Cr alloy was improved by 0.12 at. % Ag elements. The softening temperature of the Cu–0.2Cr alloy was 500~525 °C, and that of the Cu–0.2Cr–0.12Ag alloy was about 550 °C. The softening process was accompanied by the recovery, recrystallization, and coarsening of precipitates. Recovery and recrystallization occurred prior to the coarsening of precipitates. Ag elements inhibited the recrystallization of the Cu–Cr alloy and had little effect on the precipitates.
3. The creep strains of the Cu–0.2Cr and Cu–0.2Cr–0.12Ag alloys at 40 MPa and 400 °C for 50 h were 0.18% and 0.05%, respectively. The creep strain of the Cu–0.2Cr alloy was significantly greater than that of the Cu–0.2Cr–0.12Ag alloy at 500 °C. Alloy creep was accompanied by secondary precipitation of the Cr phase, dislocation slip, and recrystallization. The addition of 0.12 at. % Ag increased the creep resistance of the Cu–0.2Cr alloy mainly by hindering the increase in mobile dislocation density.

## Figures and Tables

**Table 1:** Chemical compositions of studied alloys.

Nominal Composition (at. %)	Analyzed Composition (at. %)		
	Cr	Ag	Cu
Cu–0.2Cr	0.2	/	Bal.
Cu–0.2Cr–0.12Ag	0.2	0.12	Bal.

## References

[1] Xu G.-L., Peng L.-J., Huang G.-J., Xie H.-F., Yang Z., Feng X., Yin X.-Q., Yang Z. (2019). Microstructural evolution and properties of a Cu–Cr–Ag alloy during continuous manufacturing process. Rare Met..

[2] Zhang Y., Sun H.-L., Volinsky A.A., Tian B.-H., Chai Z., Liu P., Liu Y. (2016). Characterization of the Hot Deformation Behavior of Cu–Cr–Zr Alloy by Processing Maps. Acta Met. Sin. Engl. Lett..

[3] Sun Y., Peng L., Huang G., Feng X., Xie H., Mi X., Liu X. (2021). Effect of Mg on the stress relaxation resistance of Cu–Cr alloys. Mater. Sci. Eng. A.

[4] Huang A., Wang Y., Wang M., Song L., Li Y., Gao L., Huang C., Zhu Y. (2019). Optimizing the strength, ductility and electrical conductivity of a Cu-Cr-Zr alloy by rotary swaging and aging treatment. Mater. Sci. Eng. A.

[5] Feng X., Xie H., Li Z., Mi X., Huang G., Peng L., Yang Z., Yin X. (2018). Comparison of Ag and Zr with same atomic ratio in Cu-Cr alloy. IOP Conf. Ser. Mater. Sci. Eng..

[6] Batra I., Dey G., Kulkarni U., Banerjee S. (2001). Microstructure and properties of a Cu–Cr–Zr alloy. J. Nucl. Mater..

[7] Cheng J., Yu F., Shen B. (2014). Solute clusters and chemistry in a Cu–Cr–Zr–Mg alloy during the early stage of aging. Mater. Lett..

[8] Liu Y., Li Z., Jiang Y., Zhang Y., Zhou Z., Lei Q. (2017). The microstructure evolution and properties of a Cu–Cr–Ag alloy during thermal-mechanical treatment. J. Mater. Res..

[9] Sun Y., Peng L., Huang G., Xie H., Mi X., Liu X. (2020). Effects of Mg addition on the microstructure and softening resistance of Cu–Cr alloys. Mater. Sci. Eng. A.

[10] Freudenberger J., Lyubimova J., Gaganov A., Witte H., Hickman A., Jones H., Nganbe M. (2010). Non-destructive pulsed field CuAg-solenoids. Mater. Sci. Eng. A.

[11] Xu S., Fu H., Wang Y., Xie J. (2018). Effect of Ag addition on the microstructure and mechanical properties of Cu-Cr alloy. Mater. Sci. Eng. A.

[12] Islamgaliev R.K., Sitdikov V.D., Nesterov K.M., Pankratov D.L. (2014). Structure and crystallographic texture in the Cu-Cr-Ag alloy subjected to severe plastic deformation. Rev. Adv. Mater. Sci..

[13] Yuan D., Yang B., Chen J., Chen H., Zhang J., Wang H. (2017). Upward Continuous Casting in the Manufacture of Cu-Cr-Ag Alloys: Potential for Enhancing Strength Whilst Maintaining Ductility. Met. Mater. Trans. A.

[14] Cao Y., Li Z., Zhang X., Wang Z., Qi L., Zhao H. (2019). Dynamic recrystallization behavior of upward continuous casting Cu-0.19Cr-0.1Ag alloy. Mater. Res. Express.

[15] Xu G., Mi X., Peng L., Huang G., Xie H., Yang Z., Feng X., Yin X., Xu L.G. (2019). High-temperature deformation behavior of the Cu-0.21 Cr-0.12Ag alloy made by upward continuous casting. Mater. Res. Express.

[16] Watanabe C., Monzen R., Tazaki K. (2007). Mechanical properties of Cu–Cr system alloys with and without Zr and Ag. J. Mater. Sci..

[17] Mahmudi R., Karsaz A., Akbari-Fakhrabadi A., Geranmayeh A.R. (2010). Impression creep study of a Cu–0.3Cr–0.1Ag alloy. Mater. Sci. Eng. A.

[18] Peng L.J., Mi X.J., Xie H.F., Yu Y., Huang G.J., Yang Z., Feng X., Yin X.Q. (2018). Microstructure and Properties of Cu-Cr-Zr-Ag Alloy. Mater. Sci. Forum.

[19] Wang K., Liu K.-F., Zhang J.-B. (2014). Microstructure and properties of aging Cu–Cr–Zr alloy. Rare Met..

[20] Cheng J., Shen B., Yu F. (2013). Precipitation in a Cu–Cr–Zr–Mg alloy during aging. Mater. Charact..

[21] Chbihi A., Sauvage X., Blavette D. (2012). Atomic scale investigation of Cr precipitation in copper. Acta Mater..

[22] Knights R.W., Wilkes P. (1973). Precipitation of chromium in copper and copper-nickel base alloys. Met. Mater. Trans. A.

[23] Peng L., Xie H., Huang G., Xu G., Yin X., Feng X., Mi X., Yang Z. (2017). The phase transformation and strengthening of a Cu-0.71 wt% Cr alloy. J. Alloy. Compd..

[24] Fujii T., Nakazawa H., Kato M., Dahmen U. (2000). Crystallography and morphology of nanosized Cr particles in a Cu–0.2% Cr alloy. Acta Mater..

[25] Yang W., Ji S., Wang M., Li Z. (2014). Precipitation behaviour of Al–Zn–Mg–Cu alloy and diffraction analysis from η′ precipitates in four variants. J. Alloy. Compd..

[26] Gallagher P.C.J. (1970). The influence of alloying, temperature, and related effects on the stacking fault energy. Metall. Trans..

[27] Tang N.Y., Taplin D.M.R., Dunlop G.L. (2013). Precipitation and aging in high-conductivity Cu–Cr alloys with additions of zirconium and magnesium. Met. Sci. J..

[28] Jinshui C., Bin Y., Junfeng W., Xiao X., Huiming C., Hang W. (2018). Effect of different Zr contents on properties and microstructure of Cu-Cr-Zr alloys. Mater. Res. Express.

[29] Butrymowicz D.B., Manning J.R., Read M.E. (1974). Diffusion in Copper and Copper Alloys, Part II. Copper-Silver and Copper-Gold Systems. J. Phys. Chem. Ref. Data.

[30] Adorno A.T., Beatrice C.R.S., CILENSE M., Petroni I.A., Hara A.H. (2000). Influence of silver additions on the recrystallization kinetics of the Cu-5wt.%Al alloy. Eclética Química.

